# Glucose to albumin ratio as a new predictor of postoperative pressure ulcers and hospital length of stay in geriatric hip fracture patients

**DOI:** 10.3389/fnut.2025.1639306

**Published:** 2025-09-10

**Authors:** Yazhou Liu, Ying Yang, Yuhao Li, Xiaodong Yang

**Affiliations:** ^1^Graduate School, Dalian Medical University, Dalian, China; ^2^Graduate School, China Medical University, Shenyang, China; ^3^Dandong Central Hospital, Dandong, China

**Keywords:** hip fracture, predictive indicator, glucose to albumin ratio, gar, postoperative pressure ulcers

## Abstract

**Background:**

Despite the availability of several interventions, the incidence of pressure ulcers (PU) has not significantly decreased, particularly in older adults undergoing hip fracture surgery. Therefore, there is an urgent need to develop and validate a more reliable and effective predictor to enhance the prediction accuracy for PU development in this vulnerable population.

**Methods:**

In this study, a reliable and convenient predictor of PU was developed and evaluated based on four commonly used hematological markers. The data were randomly divided into a training cohort and a validation cohort in a 7:3 ratio. The strength of the association between each predictor and the occurrence of PU was assessed using multivariate logistic regression analysis and propensity score matching (PSM). For predictors with significant associations, the receiver operating characteristic (ROC) curve and its parameters were further applied to select the best predictive model. The model was subsequently validated by a systematic evaluation of its discriminative ability, correlation, and predictive performance. Additionally, threshold analysis, subgroup analysis, and further exploration of the relationship between the GAR indicator and length of hospitalization (LOS) was conducted.

**Results:**

A total of 1,279 older adults undergoing hip fracture surgery were included in this study, with 156 (12.2%) developing PU postoperatively. Multivariate logistic regression and PSM analyses revealed a nonlinear positive correlation between GAR and postoperative PU (OR = 1.84, 95% CI: 1.44–2.35). The area under the ROC curve (AUC) for GAR was 0.72, indicating moderate predictive ability. Furthermore, each 0.1-unit increase in preoperative GAR was associated with an approximately 0.17-day increase in the length of hospitalization.

**Conclusion:**

Preoperative GAR levels are a moderate predictor of the risk of postoperative PU and LOS in older adults with hip fractures.

## Introduction

Postoperative pressure ulcers (PU) represent a prevalent and costly complication in the management of hip fractures, with reported prevalence rates ranging from 8.8 to 55% (1, 2). These ulcers significantly prolong the length of hospitalization (3) and serve as a critical indicator of poor health outcomes in older adults with hip fractures (4, 5). In older adults undergoing hip fracture surgery, the development of PU is not solely attributed to mechanical pressure, but is also influenced by underlying vulnerabilities such as diminished physiological reserve, impaired tissue repair capacity, and difficulty mobilizing postoperatively (6). Clinical guidelines emphasize the need for early mobilization within 24 h (7); however, delayed mobilization often occurs in older adults due to various factors, which can initiate a cascade of ischemic tissue damage (7).

While prolonged mechanical pressure over bony prominences remains a primary causative factor, with external pressure >32 mm Hg impairing perfusion (8–10), this explanation is insufficient in the context of geriatric hip fracture patients. In this population, the development of PU results from a combination of factors, including systemic vulnerability, chronic inflammation, malnutrition, and comorbidities (5, 6). Immobility-induced ischemia further prolongs exposure to pressure (4), while microvascular dysfunction, arising from age- and disease-related impairments in the hypoxic response, contributes to delayed tissue repair mechanisms (10, 11).

Current research primarily focuses on alleviating mechanical pressure at bony prominence sites to prevent the onset of PU (12, 13). Additionally, a study by Aline et al. has investigated the potential involvement of inflammatory responses in PU development (11). More recently, nutritional status has emerged as a significant factor influencing PU formation, garnering increasing attention in related research (14, 15). However, many of these studies exhibit methodological limitations and often lack generalizability and clinical applicability.

Considering these factors, the present study aims to investigate four well-established hematological markers that reflect systemic inflammation and nutritional status. By integrating these markers into a composite predictive index, this study seeks to develop a reliable, clinically applicable tool for early prediction of PU development and prolonged LOS, ultimately guiding early intervention strategies and improving patient management in hip fracture cases.

## Methods

### Study design and data collection

This retrospective cohort study utilized electronic medical record data of hip fracture patients treated at Dandong central hospital between January 2017 and November 2024. Baseline patient characteristics, as well as laboratory test results obtained within 48 h of admission, were systematically collected from the patients’ medical records. Blood samples were not prospectively collected for this study but were obtained as part of routine clinical care, with the laboratory results retrieved from patient records. These samples were processed and analyzed according to standard operating procedures in the hospital’s biochemical laboratory. Data collection was conducted independently by two authors (LYZ and YY), and any discrepancies were carefully examined to ensure the accuracy and consistency of the data. In line with the ethical principles outlined in the 1964 Declaration of Helsinki, the study was approved by the Institutional Review Board (IRB), and thus, no separate written informed consent was required from the participants.

### Study population

**Figure 1:**
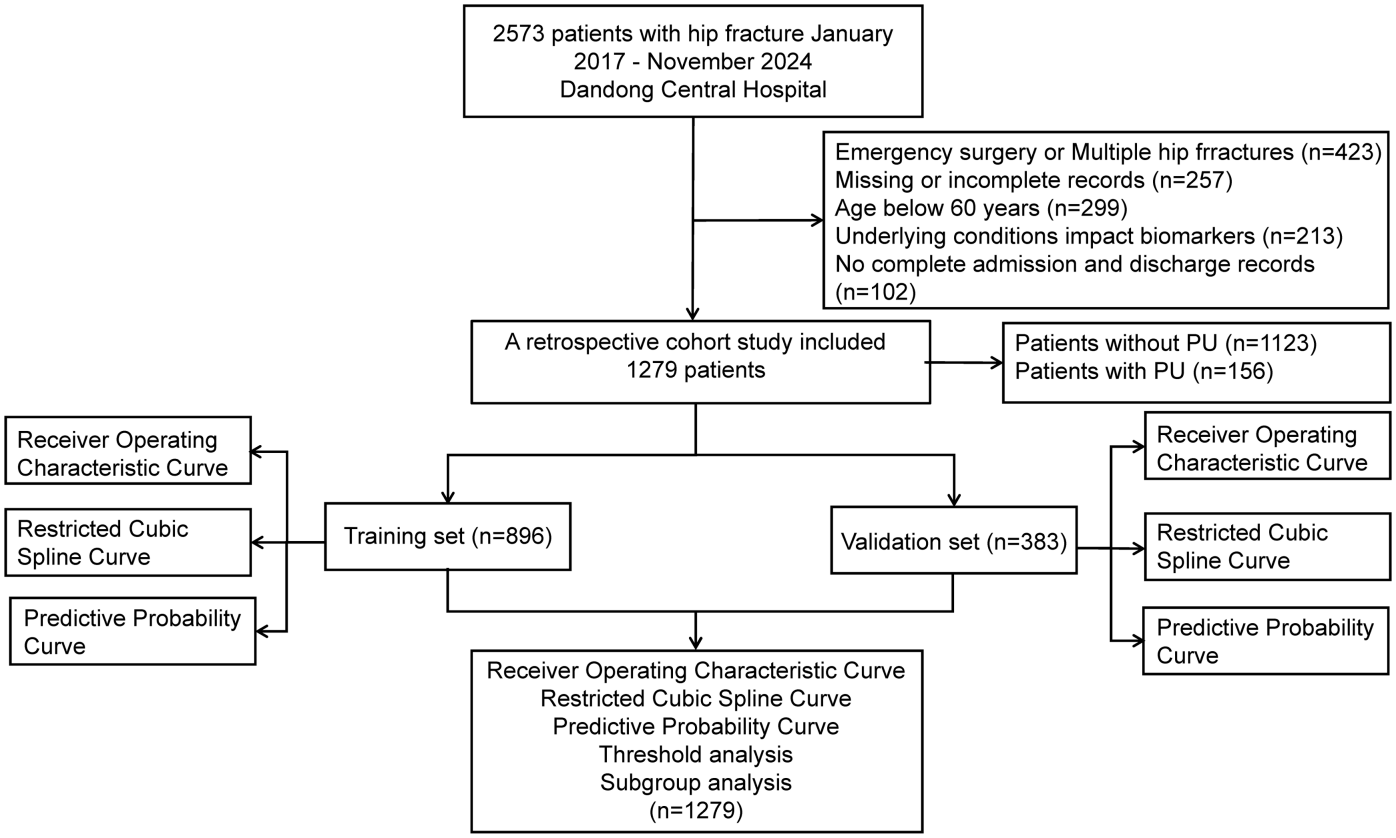
Flow diagram of enrollment.

The study population comprised patients who underwent surgical treatment for hip fractures. The exclusion criteria were as follows: (1) multiple or pathologic hip fractures; (2) age below 60 years; (3) absence of preoperative laboratory tests or incomplete electronic medical records within 48 h prior to surgery; (4) Patients who underwent emergency surgery, defined as surgeries performed for hip fractures in patients admitted through the emergency department without preoperative laboratory testing within 48 h prior to surgery, due to the urgent nature of their condition (e.g., those requiring immediate surgical intervention to address acute complications such as fractures with significant displacement or those with acute pain unmanageable by conservative means); (5) underlying medical conditions directly influencing the four hematological indices, such as infections, cirrhosis, exogenous albumin supplementation, and leukemia; (6) incomplete admission and discharge records; and (7) presence of PU upon hospital admission. The screening process is illustrated in Figure 1.

### Selection of hematological markers

In this study, we selected four hematologic markers-glucose, albumin, neutrophils, and lymphocytes-and their respective integrated composites to assess their relevance in predicting postoperative PU in hip fracture patients. These markers were chosen based on their roles in systemic vulnerability and their relevance to tissue resilience. High glucose levels reflect metabolic stress, low albumin levels indicate nutritional depletion, elevated neutrophils signal inflammation, and decreased lymphocytes reflect impaired immune function (6, 16, 17).

The Glucose-to-Albumin Ratio (GAR) and Glucose-to-Neutrophil Ratio (GNR) were calculated as follows:


GAR=Glucose(mmol/L)/Albumin(g/L)×10

GNR=Glucose(mmol/L)/Neutrophil count(×10^9/L)

These ratios combine the effects of metabolic stress, nutritional depletion, and inflammation, all key factors influencing PU risk.

### Outcome

According to the guidelines for PU management published by the European Pressure Ulcer Advisory Panel (EPUAP) (18), the assessment of PU should be conducted by the attending physician in collaboration with the nursing staff, with regular examinations of the patient’s skin condition, focusing on high-risk areas. PU are caused by ischemia and necrosis of the skin and underlying tissues due to sustained pressure, and are commonly found in pressure-prone areas, such as bony prominences. As per the EPUAP definition, any injury involving partial or complete loss of the epidermis, dermis, or both (i.e., grade 2 and above PU) is classified as a PU. Clinically, healthcare professionals create individualized treatment plans based on the patient’s presentation and PU risk assessment tools (e.g., Braden Rating Scale).

In this study, the length of stay (LOS) was defined as the period from the day of surgery to the day of hospital discharge (19).

### Covariates

Building on the risk factors identified in previous studies, we systematically extracted relevant covariates from patient medical records and organized them into four primary categories: demographic variables, comorbidity variables, surgery-related variables, and preoperative laboratory test variables. Specifically, demographic variables included age, gender, body mass index (BMI), and smoking and alcohol consumption status. Comorbidities included the American Society of Anesthesiologists (ASA) classification, dementia (defined based on medical records, including diagnosis on admission, medical history, and psychiatric consultations for cognitive impairment during the hospital stay, which were recorded in the patient’s chart), hypertension, diabetes mellitus, stroke, chronic obstructive pulmonary disease (COPD), cardiovascular disease, and cerebrovascular disease. Surgical variables included fracture type, surgical approach, surgery duration, intraoperative blood loss, and blood transfusion. Preoperative laboratory test variables included red blood cell count and hemoglobin levels.

### Statistical analysis

Based on the results of the Kolmogorov–Smirnov test, the baseline characteristics of the patients were described using the median (interquartile range) for continuous data and percentages (with corresponding counts) for categorical data, as the continuous variables were non-normally distributed. To assess statistical differences in baseline characteristics between the PU and non-PU groups, categorical data were compared using the chi-square test, while continuous data were compared using the Wilcoxon rank-sum test. The dataset was randomly divided into a training set and a validation set in a 7:3 ratio, which were used to construct the model, select the best predictors, and evaluate the predictive performance of these indicators.

The selection of predictors involved multivariate logistic regression analysis, propensity score matching (PSM) analysis, and receiver operating characteristic (ROC) curve analysis. The strength of association between predictors and PU was assessed using both multivariate logistic regression and PSM analyses, with predictors that lacked significant association with PU being excluded. Odds ratios (OR) and 95% confidence intervals (95% CI) were used to quantify these associations. Both the multivariate logistic regression and PSM analyses were designed to minimize the potential impact of bias. In multivariate logistic regression, the adjustment variables were those that were statistically significant in univariate analysis and did not exhibit multicollinearity. To detect multicollinearity, the variance inflation factor (VIF) was calculated for each variable, with a VIF greater than 10 indicating significant multicollinearity. PSM analysis employed a nearest-neighbor matching algorithm with a 1:1 ratio for all covariates, with calipers set at 0.1 standard deviations. The balance of the matched sets was assessed using the absolute standardized mean difference (SMD), with an SMD ≥ 0.10 indicating a significant imbalance between matched groups. Logistic regression analysis was then performed on the matched data to calculate the PSM-adjusted OR and 95% CI. Ultimately, the best predictor was selected based on statistical metrics such as the area under the curve (AUC), specificity, and sensitivity for each predictor.

GAR was rescaled by a factor of 10 to account for the scale effect in the logistic regression analysis, which allowed for a more stable model and a clearer interpretation of the relationship between GAR and postoperative PU.

In the validation set, ROC curves, restricted cubic spline curves, and predictive probability curves were used to evaluate the clinical predictive ability of the best predictors for PU. The threshold for the best predictive index was determined through threshold analysis, providing a reference for clinical decision-making. Additionally, generalized linear regression (GLM) analysis was conducted to explore the association between GAR and length of stay (LOS) in older adults undergoing hip fracture surgery. Finally, subgroup analyses were performed to investigate the synergistic effects of different variables and to identify which predictors are more strongly correlated with PU in specific populations. Two-sided *p*-values were used for all statistical tests, with *p* < 0.05 indicating statistical significance. Data were analyzed using IBM SPSS Statistics 26.0 and R version 4.3.1.

## Results

In this study, 2,573 electronic medical records were collected between January 2017 and November 2024. After applying the inclusion and exclusion criteria, 1,279 patients were enrolled. The results indicated that 156 patients (12.2%) developed PU postoperatively. These patients were subsequently randomized into a training cohort (896 patients) and a validation cohort (383 patients), as shown in Figure 1. The mean length of hospital stay (LOS) for the entire cohort was 10.69 days. The median age of the participants was 76 years (interquartile range [IQR]: 66.00–82.00), with 39.72% male and 60.28% female patients. Among the four hematologic markers used to construct the predictors, lymphocyte count, blood glucose levels, and albumin levels showed significant differences between the PU and non-PU groups (*p* < 0.05). Table 1 summarizes the baseline characteristics of all participants.

**Table 1:** Baseline characteristics of the patients with hip fracture.

Characteristics	Total patients (*n* = 1,279)	Patients with hip fractures		*p* value
		Non-PU (*n* = 1,123)	PU (*n* = 165)	
Demographics				
Age, years (Median, IQR)	76.00 (16.00)	75.00 (17.00)	78.00 (14.00)	0.007
Male (*n*, %)	508 (39.72%)	268 (44.22%)	154 (39.19%)	0.867
BMI ≥ 30.0 kg/m^2^ (*n*, %)	253 (19.78%)	92 (15.18%)	83 (21.12%)	0.374
Smoking (*n*, %)	218 (17.04%)	116 (19.14%)	59 (15.01%)	0.032
Alcohol (*n*, %)	148 (11.57%)	76 (12.54%)	45 (11.45%)	0.292
Comorbidities				
Dementia (*n*, %)	48 (3.75%)	21 (3.47%)	19 (4.83%)	0.157
Hypertension (*n*, %)	636 (49.73%)	243 (40.10%)	217 (55.22%)	<0.001
Diabetes (*n*, %)	297 (23.22%)	47 (7.76%)	63 (16.03%)	<0.001
Stroke (*n*, %)	332 (25.96%)	132 (21.78%)	102 (25.95%)	0.025
COPD (*n*, %)	150 (11.73%)	55 (9.08%)	53 (13.49%)	0.041
Cardiovascular diseases (*n*, %)	394 (30.81%)	162 (26.73%)	120 (30.53%)	0.271
Cerebrovascular diseases (*n*, %)	377 (29.48%)	148 (24.42%)	116 (29.52%)	0.015
Operative-related Factors (*n*, %)				
Type of fracture				0.708
Femoral neck fracture (*n*, %)	684 (53.48%)	374 (61.72%)	184 (46.82%)	
Intertrochanteric fracture (*n*, %)	521 (40.73%)	200 (33.00%)	190 (48.35%)	
Subtrochanteric fracture (*n*, %)	74 (5.79%)	32 (5.28%)	19 (4.83%)	
Type of surgery				<0.001
Total Hip Arthroplasty (*n*, %)	162 (12.67%)	91 (15.02%)	34 (8.65%)	
Hemiarthroplasty (*n*, %)	322 (25.18%)	146 (24.09%)	105 (26.72%)	
Intramedullary nail fixation (*n*, %)	416 (32.53%)	159 (26.24%)	147 (37.40%)	
Fixation with steel plate (*n*, %)	170 (13.29%)	64 (10.56%)	63 (16.03%)	
Fixation with hollow nails (*n*, %)	209 (16.34%)	146 (24.09%)	44 (11.20%)	
ASA classes ≥ III (*n*, %)	712 (55.67%)	296 (48.84%)	223 (56.74%)	0.002
Time to surgery, days (Median, IQR)	5.00 (4.00)	5.00 (4.00)	7.00 (6.00)	<0.001
Duration of surgery, hours (Median, IQR)	1.50 (0.03)	1.50 (0.83)	1.50 (0.83)	0.476
Operative blood loss, mL (Median, IQR)	130.00 (110.00)	120.00 (112.00)	170.00 (103.25)	0.398
Blood transfusion (*n*, %)	210 (16.42%)	79 (13.04%)	76 (19.34%)	0.435
Preoperative laboratory tests				
RBC count, ×109/L (Median, IQR)	3.95 (0.93)	3.96 (0.92)	3.89 (0.98)	0.53
NEU count, ×109/L (Median, IQR)	6.40 (3.30)	6.40 (3.30)	6.50 (3.10)	0.310
LYM count, ×109/L (Median, IQR)	1.20 (0.70)	1.20 (0.70)	1.15 (0.52)	0.008
HGB, g/L (Median, IQR)	121.00 (26.00)	121.00 (26.00)	120.50 (29.00)	0.595
Albumin, g/L (Median, IQR)	38.00 (6.00)	38.00 (6.00)	37.00 (7.00)	0.031
Blood glucose, mmol/L (Median, IQR)	6.10 (2.10)	6.00 (1.80)	7.30 (4.48)	<0.001

Continuous variables are presented as Median ± IQR deviation, while categorical variables are represented by numbers (percentages). IQR, Interquartile Range; BMI, Body Mass Index; COPD, chronic obstructive pulmonary disease; ASA, American Society of Anesthesiologists; RBC, Red Blood Cells; NEU, Neutrophils; LYM, Lymphocytes; HGB, Hemoglobin.

After identifying covariates significantly associated with PU through univariate logistic regression, these variables were included in the multivariate logistic regression analysis. The detailed information of the adjustment variables is provided in Supplementary Table 1. No multicollinearity was detected between the covariates (VIF < 10). The baseline characteristics of the patients before and after PSM based on GAR thresholds are presented in Supplementary Table 2. Table 2 summarizes the results of both multivariate logistic regression and PSM analyses. The final analysis revealed a complex association between GAR levels and the occurrence of postoperative PU. After adjusting for confounders, GAR was included as a continuous variable in the analysis, showing a significant positive association with the risk of postoperative PU (OR = 1.84, 95% CI: 1.44–2.35). Specifically, each 0.1-unit increase in GAR was associated with an 8.4% increase in PU risk. This indicates that higher GAR values, reflecting elevated glucose and lower albumin levels, strongly predict PU development.

**Table 2:** Unadjusted and adjusted associations between predictors and postoperative pressure ulcers.

Predictive indicator*	Data type❊	Unadjusted OR (95% CI)	*p* value	Multivariable regression adjusted OR (95% CI)	*p* value	PSM adjusted OR (95% CI)	*p* value
Neutrophils count	Continuous, Per unit increase	0.999 (0.93, 1.07)	0.983	NA	NA	NA	NA
	dichotomy, ≥4.95	1.37 (0.85, 2.21)	0.199	NA	NA	1.19 (0.67, 2.12)	0.556
Lymphocytes count	Continuous, Per unit increase	0.68 (0.47, 0.997)	0.048	0.77 (0.52, 1.14)	0.195	NA	NA
	dichotomy, ≥1.26	0.57 (0.37, 0.85)	0.007	0.62 (0.40, 0.95)	0.027	0.59 (0.37, 0.95)	0.028
Glucose	Continuous, Per unit increase	1.21 (1.14, 1.28)	<0.001	1.23 (1.14, 1.32)	<0.001	NA	NA
	dichotomy, ≥6.75	3.73 (2.47, 5.63)	<0.001	3.20 (1.99, 5.14)	<0.001	2.94 (1.61, 5.35)	<0.001
Albumin	Continuous, Per unit increase	0.94 (0.91, 0.98)	0.005	0.97 (0.92, 1.01)	0.170	NA	NA
	dichotomy, ≥37.5	0.57 (0.39,0.86)	0.007	0.75 (0.48,1.15)	0.187	0.78 (0.46,1.32)	0.351
GAR	Continuous, Per 10 increases	1.87 (1.53, 2.29)	<0.001	1.84 (1.44, 2.35)	<0.001	NA	NA
	dichotomy, ≥1.65	4.75 (2.94, 7.67)	<0.001	3.88 (2.28, 6.60)	<0.001	2.74 (1.48, 5.07)	0.001
GNR	Continuous, Per unit increase	1.74 (1.34, 2.27)	<0.001	1.72 (1.29, 2.31)	<0.001	NA	NA
	dichotomy, ≥1.295	2.67 (1.79, 3.99)	<0.001	2.35 (1.53, 3.60)	<0.001	2.72 (1.58, 4.69)	<0.001
GLR	Continuous, Per unit increase	1.05 (1.01, 1.08)	0.017	1.03 (0.996, 1.06)	0.092	NA	NA
	dichotomy, ≥7.295	2.99 (1.99, 4.47)	<0.001	2.27 (1.47, 3.51)	<0.001	2.23 (1.29, 3.85)	0.004
NLR	Continuous, Per unit increase	1.01 (0.97, 1.05)	0.724	NA	NA	NA	NA
	dichotomy, ≥4.055	1.76 (1.09, 2.83)	0.020	1.44 (0.88, 2.36)	0.145	1.50 (0.85, 2.63)	0.159
NAR	Continuous, Per unit increase	1.70 (0.17, 17.57)	0.656	NA	NA	NA	NA
	dichotomy, ≥0.175	1.37 (0.92, 2.04)	0.118	NA	NA	1.00 (0.63, 1.580)	1.000
ALR	Continuous, Per unit increase	1.003 (0.99, 1.01)	0.581	NA	NA	NA	NA
	dichotomy, ≥29.26	1.45 (0.96, 2.18)	0.076	NA	NA	1.30 (0.83, 2.05)	0.252

NA, Not Applicable; CI, Confidence Interval; OR, Odds Ratio; PSM, Propensity Scores Matching; GAR, Glucose to Albumin Ratio; GLR, Glucose to Lymphocyte Ratio; GNR, Glucose to Neutrophil Ratio; NLR, Neutrophil to Lymphocyte Ratio; NAR, Neutrophil to Albumin Ratio; ALR, Albumin to Lymphocyte Ratio.

*The highlighted red text indicates a statistically significant correlation between the predicted indicator and Postoperative pressure ulcers.

❊The dichotomy cut-off value was determined using the Youden index.

**Figure 2:**
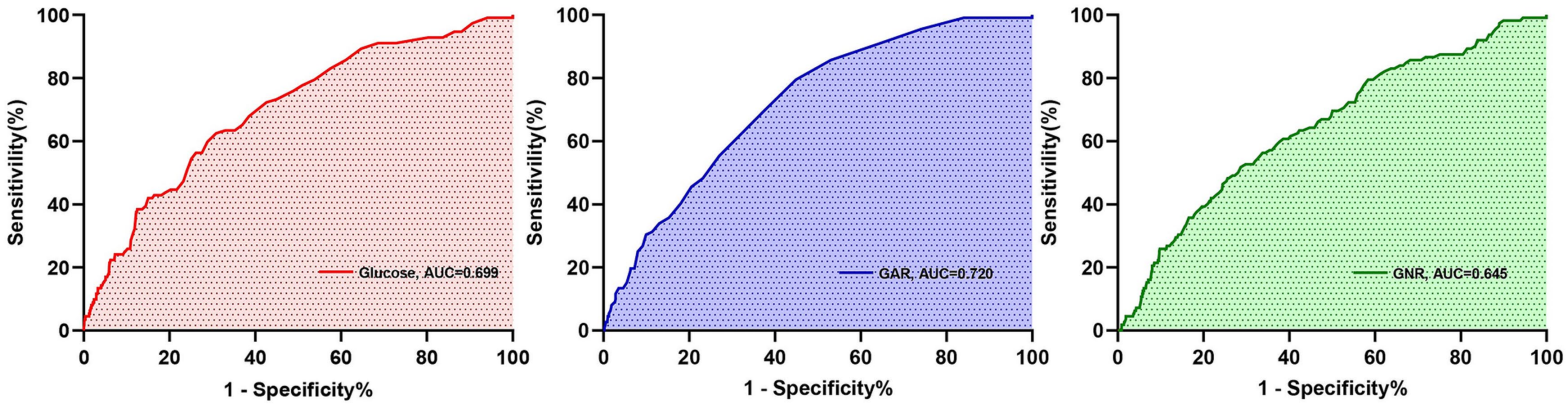
Receiver operating characteristic curve analysis for predictive indicators related to postoperative PU.

**Figure 3:**
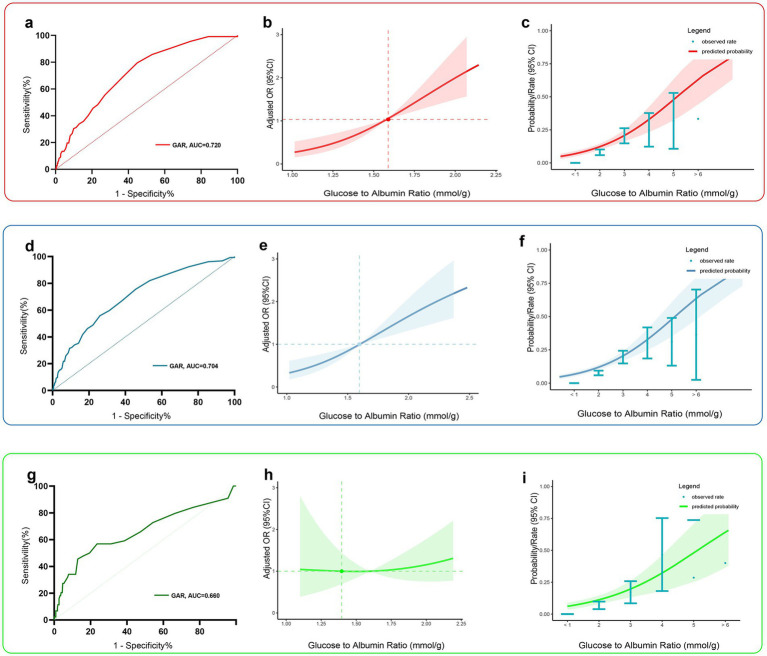
Predictive performance of the best predictor, GAR, in the training group (red), the total data set group (blue), and the validation group (green). Panels **(a, d, g)** show receiver operating characteristic curves, and panels **(b, e, h)** show restricted cubic spline curves indicating the strength of the adjusted association between preoperative GAR levels and postoperative PU. Panels **(c, f, i)** show predictive probability curves showing the predicted probability of postoperative PU at different preoperative GAR levels. Red or blue or green shaded areas indicate 95% confidence intervals.

Among all predictors, preoperative glucose levels, GAR, and GNR were significantly associated with PU development (*p* < 0.001). Among these closely related predictors, GAR exhibited the strongest predictive ability for PU in hip fracture patients (see Figure 2). ROC curve analysis revealed that GAR, as a predictor, had an AUC of 0.720, with a sensitivity of 79.5% and specificity of 55.1% (see Figure 3a). For further characterization parameters of each predictor, refer to Table 3.

**Table 3:** Assessment of the characteristic parameters of each predictive indicator.

Predictive indicator	AUC (95% CI)	SEN (%)	SPE (%)	ACC (%)	PPV (%)	NPV (%)	DeLong test *(*p* value)
GAR	0.720 (0.674, 0.766)	79.5	55.1	67.61	20.18	94.94	Reference
Glucose	0.699 (0.649, 0.752)	62.5	69.1	68.14	21.47	92.24	0.1501
GNR	0.645 (0.592, 0.701)	51.8	71.3	67.46	19.7	92.06	0.005

GAR, Glucose to Albumin Ratio; GNR, Glucose to Neutrophil Ratio; AUC, Area Under the Curve; ACC, Accuracy; SEN, Sensitivity; SPE, Specificity; PPV, Positive Predictive Value; NPV, Negative Predictive Value.

*The DeLong test utilizes the Area Under the Curve to assess whether there is a significant difference in performance between two predictive indicators.

In both the overall study population and the validation set, GAR demonstrated moderate predictive ability for PU (Figures 3d,g, AUC of 0.720 [95% CI: 0.674–0.766] and 0.660 [95% CI: 0.615–0.726], respectively). Analysis using restricted cubic spline (RCS) curves confirmed a positive correlation between preoperative GAR levels and PU risk: as the preoperative GAR level increased, the risk of postoperative PU also increased significantly (Figures 3b,e,h). The model was adjusted for all covariates to control for potential confounding factors.

Figures 3c,f,i display the predicted probability of postoperative PU in hip fracture patients at different levels of preoperative GAR. To further explore the relationship between preoperative GAR and PU, a threshold effect analysis was performed. The results showed a significant nonlinear relationship between preoperative GAR and PU (see Table 4 for details). The analysis identified GAR = 2.2 as a key inflection point: when the GAR level was below 2.2, the probability of PU increased significantly with rising GAR levels.

**Table 4:** Threshold analysis of Glucose to Albumin Ratio on Postoperative Pressure Ulcers in geriatric hip fracture patients.

Postoperative PU		Adjusted OR (95% CI)	*p* value	P for Log-likelihood ratio^a^
Postoperative PU				
GAR	Fitting by the standard linear model	1.85 (1.49, 2.29)	<0.0001	
	Inflection point:2.2			0.007
	Fitting by the two-piecewise linear model			
	ALI index<2.2	4.44 (2.26, 8.72)	<0.0001	
	ALI index≥2.2	1.48 (1.14, 1.94)	0.0038	

a Loglikelihood ratio is used to assess whether there is a statistical difference between two segmented linear models.

To further examine the relationship between preoperative GAR and LOS, we conducted a GLM regression analysis. After adjusting for confounders, the results showed that each 0.1-unit increase in preoperative GAR was associated with a significant prolongation of LOS by 0.17 days (95% CI: 0.11–0.22) (see Table 5 for details).

**Table 5:** Association between GAR and prolonged postoperative length of stay (LOS).

GAR	Model1*		Model2*	
	*β* (95% CI)	*p* value	*β* (95% CI)	*p* value
Continuous data	1.66 (1.14, 2.24)	<0.0001	2.75 (1.87, 3.82)	<0.0001
Dichotomous data	3.99 (3.37, 4.61)	<0.0001	3.66 (2.82, 4.58)	<0.0001

CI, Confidence Interval.

*Model 1, Multivariate Generalized Linear Modeling. Model 2, Multivariate generalized linear modeling after propensity score matching.

**Figure 4:**
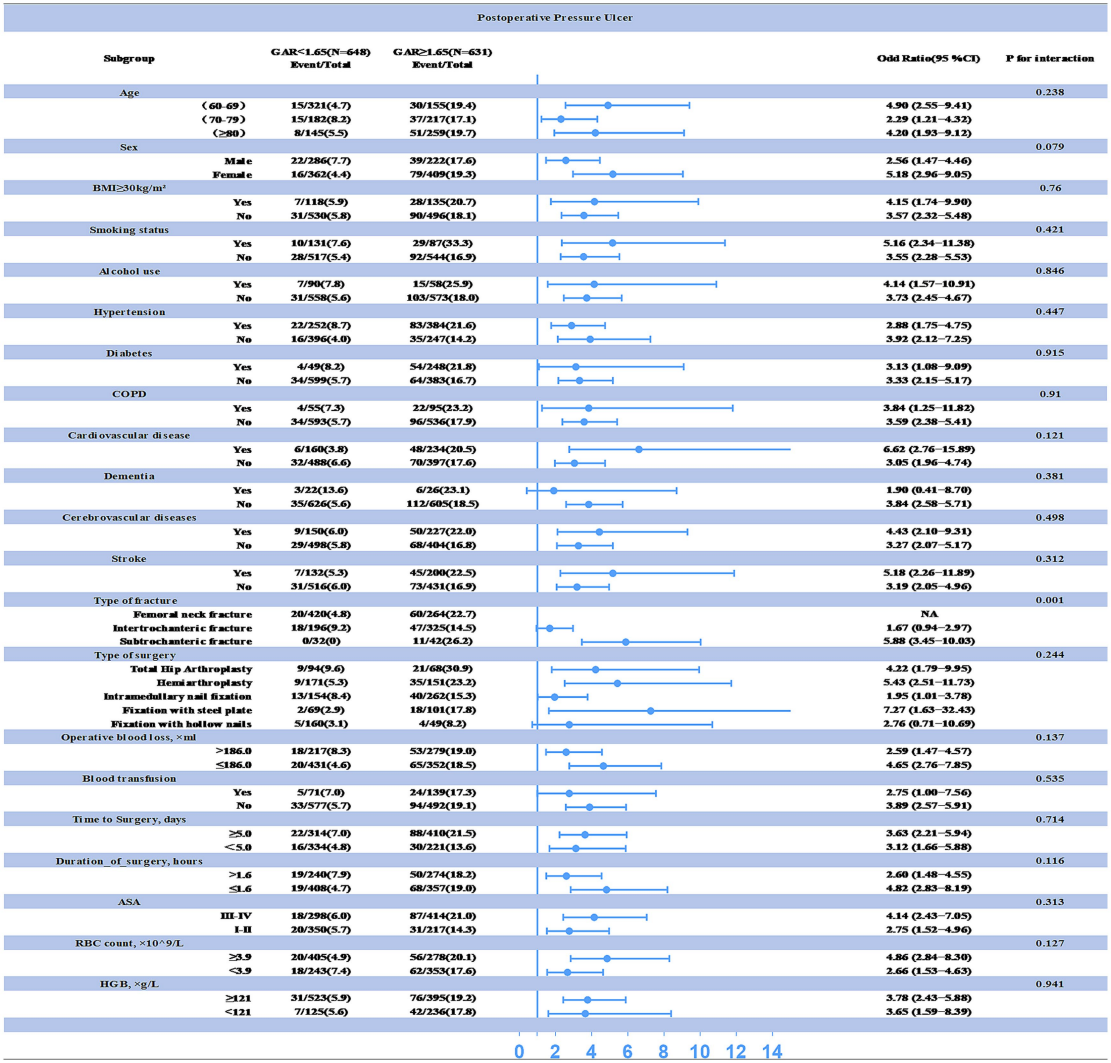
Interaction between preoperative GAR levels and other variables explored by subgroup analysis (statistical significance at *p* < 0.05). The correlation between preoperative GAR levels and postoperative PU was particularly pronounced in patients with proximal femur fractures.

Additionally, subgroup analyses were performed to assess the impact of other covariates on the relationship between preoperative GAR levels and PU (see Figure 4). The results revealed a significant interaction between fracture type, preoperative GAR level, and PU incidence (all interaction *p*-values < 0.05). Specifically, patients with proximal femur fractures exhibited a higher incidence of PU at the same preoperative GAR level. Therefore, clinicians should give special attention to elevated GAR levels when managing these patients, as this factor is strongly associated with an increased risk of postoperative PU.

## Discussion

PU remain a significant clinical challenge, particularly in frail older adults undergoing hip fracture surgery, where the incidence is high and outcomes are often poor (20). This persistent issue is largely attributed to the multifactorial nature of PU development, driven by a complex interplay of metabolic stress, nutritional depletion, impaired tissue resilience, and frailty (6, 11–15). The glucose-albumin ratio, which integrates metabolic stress (elevated blood glucose) and nutritional depletion (hypoalbuminemia), provides a novel and valuable tool for assessing PU risk in this vulnerable population, as both factors significantly impair tissue healing capacity.

This retrospective study systematically investigates hematological indicators of inflammation and nutritional status, both of which are closely linked to PU development. We identified key predictors most strongly associated with the occurrence of postoperative PU in older adults undergoing hip fracture surgery, thereby providing clinicians with a reliable and easily monitored predictive tool for PU risk. Our findings demonstrate an independent correlation between preoperative GAR levels and PU occurrence in hip fracture patients, with higher GAR levels significantly increasing the risk of PU development. Notably, the association between preoperative GAR levels and PU occurrence was even more pronounced in the proximal femur fracture subgroup, which is typically associated with frailty due to osteoporosis and other geriatric factors. ROC curve analysis revealed that the optimal GAR threshold was 1.65, with an AUC of 0.72, proving effective in predicting the risk of postoperative PU in hip fracture patients. However, the specificity of 55.1% suggests a relatively high false-positive rate, which may limit the clinical applicability of GAR as a stand-alone predictor. A higher false-positive rate could result in unnecessary interventions or monitoring for patients who may not develop PU, leading to increased healthcare costs and resource utilization. To address this limitation, we suggest that GAR could be used in combination with other clinical screening tools, such as the Braden Scale or Norton Scale, which evaluate additional factors like sensory perception, mobility, and friction/shear. By combining GAR with these tools, clinicians could improve specificity and reduce the number of false positives, enhancing the overall accuracy of PU risk prediction. Moreover, incorporating other clinical assessments, such as patient frailty or nutritional assessments, could further refine the predictive model and help guide targeted interventions. These findings underscore the potential of preoperative GAR levels as a predictor of PU risk. Additionally, threshold analysis provides clinicians with a critical inflection point of 2.2 for preoperative GAR levels, which can help enhance clinical vigilance.

Numerous studies have demonstrated that elevated blood glucose levels significantly increase the risk of developing surgery-related PU in patients (21, 22). Hip fracture patients are typically mobilized within 24 h of surgery, in accordance with current best practices, which significantly reduces the risk of immobility-related complications, including PU. However, those who are not mobilized early due to severe frailty or other medical conditions remain at a heightened risk of PU development (7). The stress associated with hip fractures and subsequent surgeries often leads to elevated blood glucose, which, in turn, contributes to delayed wound healing and an increased risk of PU development (23, 24). Hyperglycemia impairs leukocyte function, particularly during the inflammatory phase of wound healing, by inhibiting neutrophil migration, adhesion, and cytokine release (25). This compromises the immune response and prolongs the inflammatory phase, further delaying PU healing.

Moreover, hyperglycemia interferes with collagen deposition by impairing collagen synthesis and fibroblast function (26). These alterations result in delayed wound healing and increased susceptibility to ischemic necrosis, particularly at sites of sustained pressure, such as bony prominences (27, 28). Hyperglycemia also induces oxidative stress and the formation of advanced glycosylation end products (AGEs), which exacerbate tissue damage, impair microcirculation, and worsen ischemia in pressure ulcer-prone areas (29–32). This impaired tissue repair process, further exacerbated by hyperglycemia, highlights the critical need for better metabolic management in frail patients to mitigate PU development.

In addition to metabolic stress, nutritional status plays a critical role in PU development (33, 34). Malnutrition, on one hand, results in a reduction of connective tissue, thereby weakening the skin’s resistance to external pressure and lowering tissue tolerance, which in turn increases the risk of PU formation (35). In malnourished individuals who develop severe PU, the wound healing process is often significantly delayed. Among patients with hip fractures, malnutrition is particularly prevalent, rendering this population at higher risk for PU development (36). On the other hand, undernutrition is typically accompanied by a reduction in adipose tissue, diminishing the cushioning and protective functions over bony prominences. In addition, conditions such as generalized weakness, dehydration, and edema are frequently observed in malnourished patients, collectively impairing the skin’s barrier function, reducing mobility, and compromising immune defenses, thereby further elevating the risk of PU occurrence (37–40).

Serum albumin is widely used as a biomarker of nutritional status and plays a vital role in maintaining tissue integrity during mechanical loading (41–43). Low serum albumin levels are strongly associated with PU risk, as they reflect both reduced tissue repair capacity and impaired resistance to pressure-induced tissue damage (44, 45).

Frailty is a multidimensional syndrome characterized by reduced physiological reserve and increased vulnerability (6), often seen in older adults, particularly those undergoing hip fracture surgery (46). It encompasses a combination of factors, including impaired mobility, poor nutritional status, and weakened immune responses (47–49). Although frailty was not directly assessed in this study, it has been proposed as a potential unmeasured confounder influencing the relationship between GAR and postoperative PU risk. Frailty is prevalent among older adults with hip fractures and has been linked to poorer surgical outcomes and an increased susceptibility to complications, including PU. For instance, a recent meta-analysis demonstrated that frailty was associated with poor postoperative outcomes following hip fractures (50), and studies by Kistler et al. (51) and Pizzonia et al. (52) showed associations between frailty and short- and long-term hip fracture outcomes, respectively. However, due to the absence of formal frailty measures in our dataset, we were unable to draw definitive conclusions about its role in PU development. Future studies should explicitly assess frailty using validated scales, such as the Frailty Phenotype or the Clinical Frailty Scale, to gain a deeper understanding of how frailty interacts with metabolic and nutritional factors in predicting PU. This approach would enable more targeted interventions and enhance the accuracy of predictive models for PU development in frail patients.

In conclusion, this study comprehensively analyzed the role of GAR in predicting the development of PU in older adults undergoing hip fracture surgery. By combining hyperglycemia and hypoalbuminemia, the GAR provides a new, clinically relevant predictor that integrates metabolic and nutritional factors that are important for tissue healing. GAR not only facilitates the identification of at-risk patients but also lays the foundation for tailored interventions, which should be based not solely on objective indicators but also on individualized care. Future research should further investigate the direct role of frailty in PU risk and explore how GAR can be incorporated into clinical practice to improve the management and outcomes of older adults undergoing hip fracture surgery.

### Limitations

This study has several limitations that warrant transparent acknowledgment. First, as a retrospective analysis, it is subject to inherent biases, despite our efforts to minimize these through the application of appropriate statistical methods. Second, the investigation primarily focused on hematologic markers as predictors of postoperative PU, without incorporating comparative analyses of other potential risk factors. This limitation may have constrained the identification of more clinically valuable predictors. Third, while the GAR index showed promise, its specificity in identifying high-risk PU patients was limited to 55.1%, potentially resulting in misclassification. Nevertheless, considering the multifactorial etiology and complex pathophysiology of PU development, achieving standardized predictive performance from a single biomarker remains a significant challenge. Finally, the single-center nature of the study and the relatively homogeneous patient population may restrict the generalizability of the findings to broader clinical settings.

## Conclusion

Preoperative GAR levels are a reliable predictor of postoperative PU development in older adults undergoing hip fracture surgery, exhibiting a significant nonlinear dose–response relationship, particularly in those with proximal femur fractures. Specifically, each 0.1-unit increase in the GAR index was associated with an 8.4% increase in the risk of PU development. This relationship is reflective of the combined effects of elevated glucose levels and lower albumin levels. Furthermore, preoperative GAR levels were also correlated with patients’ length of stay, with a notable increase of 0.17 days in LOS for every 0.1-unit rise in preoperative GAR. In conclusion, timely medical intervention and precautionary measures are essential when preoperative GAR levels fall within the range of 1.65 to 2.2.

## Data Availability

The original contributions presented in the study are included in the article/Supplementary material, further inquiries can be directed to the corresponding author.
