# Supplementary material for: Glucose to albumin ratio as a new predictor of postoperative pressure ulcers and hospital length of stay in geriatric hip fracture patients
Source: Front Nutr. 2025 Sep 10;12:1639306. doi: 10.3389/fnut.2025.1639306 (PMC12457409; doi:10.3389/fnut.2025.1639306)
Supplement: Supplementary file 1 [file Table_1.DOCX]

# **Appendix:**

**e-Table1 Multivariate analysis for Postoperative Pressure Ulcers.**

**e-Table2 Characteristics of patients before and after propensity score matching based on GAR**

**e-Table1 Multivariate analysis for Postoperative Pressure Ulcers.**

| Characteristics | Collinearity test | Univariate | | Multivariate | |
| --- | --- | --- | --- | --- | --- |
|  | VIF | OR (95% CI) | p value | OR (95% CI) | p value |
| Demographics | | | | | |
| Age, years | 1.665 | 1.03(1.01,1.05) | 0.008 | 1.01(0.99,1.04) | 1.01 |
| Male | 1.465 | 0.995(0.66,1.49) | 0.979 | NA | NA |
| BMI ≥ 30.0 kg/m² | 1.088 | 1.07(0.66,1.74) | 0.778 | NA | NA |
| Smoking | 1.571 | 1.54(0.95,2.48) | 0.079 | NA | NA |
| Alcohol | 1.536 | 1.09(0.60,1.99) | 0.784 | NA | NA |
| Comorbidities | | | | | |
| Hypertension | 1.285 | 2.27(1.49,3.45) | <0.001 | 1.83(1.16,2.87) | 0.009 |
| Diabetes | 1.436 | 2.02(1.33,3.08) | 0.001 | 1.58(1.01,2.46) | 0.043 |
| COPD | 1.107 | 1.56(0.91,2.67) | 0.108 | NA | NA |
| Cardiovascular | 1.203 | 1.20(0.79,1.83) | 0.384 | NA | NA |
| Dementia | 1.046 | 1.00(0.34,2.91) | 0.999 | NA | NA |
| Cerebrovascular diseases | 6.482 | 1.44(0.95,2.17) | 0.088 | NA | NA |
| Stroke | 6.428 | 1.40(0.91,2.15) | 0.122 | NA | NA |
| Operative-related Factors | | | | | |
| ASA (≥III VS ＜III) | 1.266 | 1.75(1.15,2.66) | 0.009 | 1.22(0.77,1.93) | 0.399 |
| Type of fracture | 1.557 | 0.78(0.57,1.08) | 0.131 | NA | NA |
| Type of surgery | 1.345 | 0.76(0.65,0.90) | 0.001 | 1.10(1.05,1.14) | <0.001 |
| Operative blood loss, ml | 1.908 | 1.00(0.999,1.00) | 0.480 | NA | NA |
| Blood transfusion | 1.449 | 1.34(0.81,2.20) | 0.251 | NA | NA |
| Time to surgery, days | 1.137 | 1.09(1.05,1.14) | <0.001 | 0.76(0.51,1.11) | 0.157 |
| Duration of surgery, hours | 1.382 | 1.11(0.89,1.39) | 0.357 | NA | NA |
| Preoperative Laboratory Tests | | | | | |
| RBC count, ×109/L | 6.823 | 0.75(0.56,1.00) | 0.053 | NA | NA |
| NEU count, ×109/L | 1.131 | 0.999(0.93,1.07) | 0.983 | NA | NA |
| LYM count, ×109/L | 1.146 | 0.68(0.47,0.997) | 0.048 | 0.77(0.52,1.14) | 0.195 |
| HGB, g/L | 6.864 | 0.99(0.98,1.00) | 0.082 | NA | NA |
| Albumin, g/L | 1.548 | 0.94(0.91,0.98) | 0.005 | 0.97(0.92,1.01) | 0.17 |
| Blood glucose, mmol/L | 1.485 | 1.21(1.14,1.28) | <0.001 | 1.23(1.14,1.32) | <0.001 |
| Glucose to Albumin Ratio | 1.483 | 1.87(1.53,2.29) | <0.001 | 1.84(1.44,2.35) | <0.001 |
| Glucose to Lymphocytes Ratio | 1.140 | 1.05(1.01,1.08) | 0.017 | 1.03(0.996,1.06) | 0.092 |
| Glucose to Neutrophils Ratio | 1.099 | 1.74(1.34,2.27) | <0.001 | 1.72(1.29,2.31) | <0.001 |
| Neutrophils to Lymphocytes Ratio | 1.120 | 1.01(0.97,1.05) | 0.724 | 0.994(0.95,1.04) | 0.779 |
| Neutrophils to Albumin Ratio | 1.160 | 1.70(0.17,17.57) | 0.656 | 0.42(0.03,6.37) | 0.531 |
| Albumin to Lymphocytes Ratio | 1.050 | 1.003(0.99,1.01) | 0.581 | 1.001(0.99,1.01) | 0.827 |

NA, Not Applicable; CI, Confidence Interval; OR, Odds Ratio; VIF, Variance Inflation Factor; BMI, Body Mass Index; ASA, American Society of Anesthesiologists; RBC, Red Blood Cells; NEU, Neutrophils; LYM, Lymphocytes; HGB, Hemoglobin.

**e-Table2 Characteristics of patients before and after propensity score matching based on GAR**

| **Characteristics** | **Before matching** | | | **After matching** | | |
| --- | --- | --- | --- | --- | --- | --- |
|  | **GAR<1.65 (n=455)** | **GAR≥1.65**  **(n=441)** | **SMD** | **GAR<1.65 (n=223)** | **GAR≥1.65**  **(n=223)** | **SMD** |
| Demographics | | | | | | |
| Age, years | 75.00(17.00) | 78.00(14.00) | 0.650 | 77(14) | 77(13) | 0.053 |
| Male | 268 (44.22%) | 154 (39.19%) | 0.116 | 86 (38.57%) | 86 (38.57%) | <0.001 |
| BMI (≥30.0 kg/m²) | 92 (15.18%) | 83 (21.12%) | 0.010 | 38 (17.04%) | 42 (18.83%) | 0.047 |
| Smoking | 116 (19.14%) | 59 (15.01%) | 0.157 | 23 (10.31%) | 24 (10.76%) | 0.061 |
| Alcohol | 76 (12.54%) | 45 (11.45%) | 0.205 | 86 (38.57%) | 86 (38.57%) | 0.015 |
| Comorbidities | | | | | | |
| Dementia | 21 (3.47%) | 19 (4.83%) | 0.006 | 12 (5.38%) | 9 (4.04%) | 0.063 |
| Hypertension | 243 (40.10%) | 217 (55.22%) | 0.496 | 119 (53.36%) | 128 (57.40%) | 0.081 |
| Diabetes | 47 (7.76%) | 63 (16.03%) | 0.793 | 36 (16.14%) | 39 (17.49%) | 0.036 |
| Stroke | 132 (21.78%) | 102 (25.95%) | 0.223 | 66 (29.60%) | 64 (28.70%) | 0.020 |
| COPD | 55 (9.08%) | 53 (13.49%) | 0.230 | 32 (14.35%) | 29 (13.00%) | 0.039 |
| Cardiovascular diseases | 162 (26.73%) | 120 (30.53%) | 0.303 | 79 (35.43%) | 74 (33.18%) | 0.047 |
| Cerebrovascular diseases | 148 (24.42%) | 116 (29.52%) | 0.237 | 76 (34.08%) | 72 (32.29%) | 0.038 |
| Operative-related Factors | | | | | | |
| Type of fracture |  |  | 0.470 |  |  | 0.007 |
| Femoral neck fracture | 374 (61.72%) | 184 (46.82%) |  | 13 (5.83%) | 11 (4.93%) |  |
| Intertrochanteric fracture | 200 (33.00%) | 190 (48.35%) |  | 91 (40.81%) | 94 (42.15%) |  |
| Subtrochanteric fracture | 32 (5.28%) | 19 (4.83%) |  | 119 (53.36%) | 118 (52.91%) |  |
| Type of surgery |  |  | 0.094 |  |  | 0.075 |
| Total Hip Arthroplasty | 91 (15.02%) | 34 (8.65%) |  | 24 (10.76%) | 23 (10.31%) |  |
| Hemiarthroplasty | 146 (24.09%) | 105 (26.72%) |  | 73 (32.74%) | 65 (29.15%) |  |
| Intramedullary nail fixation | 159 (26.24%) | 147 (37.40%) |  | 67 (30.04%) | 69 (30.94%) |  |
| Fixation with steel plate | 64 (10.56%) | 63 (16.03%) |  | 30 (13.45%) | 34 (15.25%) |  |
| Time to surgery, days | 5.00(4.00) | 7.00(6.00) | 0.377 | 5(4) | 5(3) | 0.037 |
| Duration of surgery, hours | 1.50(0.83) | 1.50(0.83） | 0.186 | 1.50(0.03) | 1.50(0.75） | 0.028 |
| Operative blood loss, ml | 120.00（112.00） | 170.00(103.25) | 0.165 | 150（104） | 150（100） | 0.031 |
| Blood transfusion | 79 (13.04%) | 76 (19.34%) | 0.291 | 41 (18.39%) | 40 (17.94%) | 0.012 |
| ASA (≥III VS ＜III) | 296 (48.84%) | 223 (56.74%) | 0.402 | 143 (64.13%) | 133 (59.64%) | 0.092 |
| Preoperative Laboratory Tests | | | | | | |
| RBC count, ×10^9^/L | 3.96(0.92) | 3.89(0.98) | 0.531 | 3.95(0.80) | 3.99(0.98) | 0.013 |
| HGB, g/L | 121.00(26.00) | 120.50(29.00) | 0.507 | 120(5) | 121(27) | 0.019 |

Continuous variables are presented as mean ± standard deviation, while categorical variables are represented by numbers (percentages).

SMD, Standardized Mean Difference; CI, Confidence Interval; OR, Odds Ratio; BMI, Body Mass Index; COPD, chronic obstructive pulmonary disease; ASA, American Society of Anesthesiologists; RBC, Red Blood Cells; HGB, Hemoglobin.
